# The Doctrine of the Mean and Doctor–Patient Relationship: Proposal for the Doctor-Seeking-the-Mean Model

**DOI:** 10.1007/s11673-025-10476-x

**Published:** 2025-09-29

**Authors:** Atsushi Asai, Hua Xu, Motoki Ohnishi

**Affiliations:** 1 Department of Medical Ethics, Tohoku University Graduate School of Medicine, 980-8575, Address 2-1, Seiryocho, Aoba-ku, Sendai, Miyagi Prefecture Japan; 2 Department of Health and Welfare Public Policy, Laboratory of Public Health, Aomori University of Health and Welfare Graduate School of Health Science, 58-1 oaza hamadate aza mase Aomori, Aomori, 030-8505 Japan

**Keywords:** Doctor–patient relationship, Confucius, The doctrine of the Mean, Goals of Medicine, Special human relationships, Everyday ethics

## Abstract

Our experience in day-to-day medical practice suggests that the nature of the doctor–patient relationship (DPR) is considerably influenced by the attitudes of the individual patient and doctor. The DPR will also be significantly influenced by the social environment, including the healthcare system in which it develops. In addition, cultural influences on the DPR and its overriding ethical principles cannot be ignored. Moreover, the DPR cannot escape the influence of various coincidences. We argue that it is preferable that a doctor-seeking-the-Mean model be practiced, whereby doctors do not treat their patients in a uniform, normative, and skilful manner but rather try to achieve the Mean in key aspects of the DPR—which is highly individualized, changeable over time, and subject to social trends and chance. For Confucius, the Mean is a virtue that enables one to respond flexibly to changing circumstances and realize one’s goals without excess or deficiency; it is an attempt to fully understand all the diverse and conflicting views and positions before making the highest quality decision that best suits one’s need to achieve the goals at any given moment. We believe that the doctor-seeking-the-Mean model can fully fulfil the role of a platform for realizing the goals of healthcare.

## Introduction

Various models and classifications of the doctor–patient relationship (DPR) have been proposed, as well as views on the most appropriate DPR model and decision-making model (Yoneura 2023). Discussions on overriding principles in the DPR and practical issues in decision-making have also been presented (Childress and Childress 2020). However, doctor–patient conflicts in clinical settings are constant, and patients and their families can be hurt by inappropriate attitudes of doctors, or conversely, many doctors can be violated and victimized by patients and their families (Xu et al. 2024). In addition, the impact of introduction of medical artificial intelligence (AI) on the DPR has yet to be clarified (Asai et al. 2019).

The DPR is likely to be highly influenced by the personalities and attitudes of the individual patient and doctor, while it is also strongly affected by the healthcare system, major social trends and events, developments in medical technology, and regional and cultural factors. In daily practice, the DPR is influenced by various coincidences. We also believe that the DPR is subject to effects of the volatile, uncertain, complex, and ambiguous (VUCA) medical practice of today (Hamid 2019). Furthermore, we would claim that the DPR itself is more complex than the ordinary human relationship.

Given the above, in this paper, we argue that doctors should adopt attitudes and behaviours aimed at achieving the Mean in the DPR. In particular, we argue that the doctrine of the Mean proposed by the ancient Chinese philosopher Confucius is of great significance in the context of the complex, highly individualized DPR which can change over time (Confucius 1993; Confucius 2016). However, we hold a different view from Confucius regarding the goal of the Mean and clarify that the goal of the Mean in this paper is the realization of the purposes of medicine. We argue that the DPR established as a result of the doctor-seeking-the-Mean model, in which doctors aim to achieve the Mean, provides an appropriate platform for the realization of the purposes of medicine.

At the outset, we will describe the Mean in Confucius’ *Doctrine of the Mean* to distinguish it from the ordinary “mean.” The Mean can be expressed as the “Golden mean.” It should be noted that, although the importance of various health professionals other than doctors in contemporary medical practice is well recognized, this paper only focuses on the relationship between a patient and doctor in order to avoid complicating the discussion and to clarify the importance of aiming to achieve the Mean. In addition, given that the DPR is deployed in all aspects of medical care, from outpatient care to inpatient treatment and home healthcare, the following discussion on medical care, the DPR, and the Doctor-Seeking-the-Mean Model will take on various forms, starting from a simple relationship between a patient and a doctor.

In the following, we will describe the DPR as a complex relationship that is highly individualized, changeable over time, and is under the influence of social trends and chance. We will also elaborate on the Confucian Doctrine of the Mean, characterizing it by its goal-oriented nature, uniqueness, timely flexibility, comprehensiveness, humility, realistic nature, and difficult-to-achieve nature. The aim of the Mean, in our view, is the realization of the goals of medicine to eliminate and alleviate patient suffering. Then, we will argue from various perspectives that the Confucian Doctrine of the Mean has utility in the DPR.

According to Tamblyn, a contemporary writer, “Confucius is one of our very best thinkers and a model for living a self-aware and virtuous life” (Tamblyn 2016), i–ii). *The Doctrine of the Mean (Zhong Yong)* is regarded as an essential text of the Confucian ethics (Suh 2020), and as Tamblyn claims, “Its purpose is to show how the golden way is the means to gain perfect virtue, and that following the heavenly instructions of the Way will lead to the virtuous path trodden by others before, including Confucius” (Tamblyn 2016), i–ii). The Mean (*zhong-yong*) refers to taking a middle path in life, avoiding extremes. It is a virtue praised by Confucius and has long been respected as a tradition of Confucianism (Kanaya 1998). Suh also wrote, “the Confucian ideal of the *zhong-yong* society presupposes that the current society is not perfect and that humans are not perfectly rational decision-makers. There is no perfect agent who is in the state of equilibrium at all times. Neither is there a perfect society where all individuals are in harmony with everything else, which keeps changing over time. Nevertheless, Confucius throughout *Zhong Yong* (*The Doctrine of the Mean*) preaches that humans must try to keep practicing *zhong-yong* to make the world move towards perfection” (Suh 2020, 65).

## Change in Normative Models of the DPR

Various DPR model classifications have been published from the ethical, social, and legal perspectives, as well as in terms of decision-making styles (Yoneura 2023). Some models have used metaphors to describe the DPR, such as the parent–child relationship or general human relationship, while others liken it to relationships between parties in other professions. Some model classifications have focused on the information-sharing and decision-making roles of doctors and patients, while others have taken into account the equality of their relationship and contract (Szasz and Hollender 1956; Veatch 1972; Emmanuel and Emmanuel 1992; Higuchi 1998). The importance of fiduciary duty, friendship, and decision-making support in the DPR has also been noted. Looking historically at the aspects of decision-making favoured from a normative perspective, it can be said that the DPR model has undergone a transition from a paternalistic model to a patient-informed choice model, and then to a shared decision-making (SDM) model, with a critique of paternalism, an emphasis on patient self-determination, and recognition of the importance of decision-making support (Childress and Childress 2020; Yoneura 2023). In recent years, the deliberative model, which is suitable for realizing SDM between patients and doctors, has become the preferred model (Ahuja 2019).

The above typology and categorization are very significant in that they have identified from a medical-ethical perspective the advantages and problems of specific models, as well as important concepts involved. At the same time, a diverse range of DPR modes has existed over a period of about half a century. From an ethically neutral standpoint, it would not be wrong to say that the spectrum of DPR typologies or models has been expanding. Strictly speaking, it is unclear which DPR model prevails in contemporary medical practice worldwide. Previous studies suggested that only a small minority of patients prefer to leave decisions to doctors or to make decisions on their own, and that the majority prefers to make decisions in consultation with a doctor (Levinson et al. 2005; Murry et al. 2007; Japan Medical Association Research Institute for Policy and Practice 2017; Lo 2020; Yoshida 2020). However, these data only suggest general trends in a few countries and do not provide information on the current state of DPRs and decision-making models in all countries, nor can the statistics suggest the state of individual DPRs; it is obviously not possible to determine a normative DPR model from the descriptive ethical situation regarding the DPR, but descriptive diversity may indicate a high degree of acceptability of normative diversity.

## Various Matters Affecting the DPR

In the following, we suggest that various matters affect the DPR and decision-making. First, we must consider the individual nature of the DPR. One doctor stated, “the unique doctor treats a unique patient, whom he or she comes to understand and care for in a unique way” (Loxterkamp 2023, 467). Lo, a contemporary doctor-ethicist, also claims that there are variations in the DPR in medical practice and that the question arises as to what kind of DPR is most appropriate for a particular doctor, patient, and clinical situation (Lo 2020). The particularity of the DPR that develops between a patient and doctor would depend on their individual temperament, personality, and mindset, as well as mutual compatibility (Asai, Okita, and Bito 2021; Asai, Okita, and Bito 2022). The life and illness experiences of both parties, their ages, the involvement of the patient’s family, and the cognitive biases of the people involved are also relevant. Another doctor stated,

… as I have aged, I have grown more fond of trying to connect to patients on a level that is human, physician to patient. The more rewarding puzzle now is negotiating a human interaction in an increasingly impersonal environment … I am a different doctor than I was 30 years ago. (Kellerman 2018, 12)

Patients’ decision-making and attitudes toward doctors may also change depending on the stage of disease they are in (Yoshida 2020).

One qualitative study of doctors found that the DPR is unique and multifaceted in nature, encompassing professional, personal, and business-transactional dimensions. That study also mentioned the possibility of establishing a friendship and affection through the DPR (Thomas, Best, and Mitchell 2020). Interpersonal factors including the doctor’s commitment and communication skills, the doctor’s emotional state (relaxed and happy, tired, or stressed, or cynical and burn-out), and the patient’s openness to the doctor also influence the DPR (Thomas, Best, and Mitchell 2020).

The DPR is likely to be affected by changes in social conditions, and the introduction of medical AI into healthcare settings will have a profound impact. It is unclear what the future relationship between doctors, patients, and AI will be, when sophisticated medical AI is introduced, although there is much speculation (Asai et al. 2019; Cartolovni, Malesevic, and Poslon 2023). The DPR will also be significantly affected by the commercialization of healthcare, the health insurance system and its quality, and the degree of medicalization of society. For example, the way doctors interact with patients has changed, particularly with the introduction of electric medical records, where the computer is now the third person in the room, often sitting between the doctor and patient (Loxterkamp 2023). However, the impact of medical AI, which is heavily involved in diagnosis and treatment selection, on the DPR will not be comparable to that of electric medical records. In a sense, the DPR is a multi-layered relationship involving doctors, patients, families, AI, hospital policies, access to healthcare, and the healthcare system.

The DPR described above is mainly a two-party relationship between a patient and doctor; however, in some cultures, the family may be just as important a party as the patient, and healthcare may develop into a three-party relationship involving the patient, family, and doctor, with different overriding ethical principles (Asai, Okita, and Bito 2021; Asai, Okita, and Bito 2022). Due to differences in individual and family relationships, some patients may want to be briefed by the doctor together with their family members, while other patients may prefer seeing their doctors alone.

In certain cultures, patient-informed choice may be the principle and paternalism the exception, while in other cultures, paternalism may be the principle. In other words, the possibility that principles and exceptions may be reversed depending on cultures cannot be ruled out. The DPR is also influenced by concepts such as the awareness of rights, e.g., individual human rights, that have arisen through historical changes in society. It will also be affected by the extent to which these concepts are widespread in human society. For example, in human societies where the concept of the individual has not been clearly established, it is unlikely that a DPR that places the highest value on patient autonomy will exist.

We believe that the influencing factors and individualization involved in the DPR discussed so far in this section cannot escape the influence of chance. From a larger perspective, we can say that the medical situation and the DPR that would-be patients experience differ depending on which country they happen to be born in and at what time period, as well as the country’s healthcare system. The DPR that the patient is involved in would also differ by healthcare cost, available healthcare resources, business of the doctor, extent of the use of medical AI, and robustness of the healthcare insurance system. The mode of DPR can also be affected by prevailing medical ethical principles at the time and the place that the patient happens to be. Significant differences in all of them can happen by chance. We believe that these coincidental factors have a significant impact on the resulting DPR mode.

In modern day-to-day life, when a patient visits a medical institution, he or she develops a DPR that may differ depending on who the doctor in charge of the consultation is. The doctor on Monday may be a hardcore paternalist, while the doctor on Tuesday may be a self-determination fundamentalist. The DPR can also vary depending on the patient’s parentage, how he or she was brought up, and what personality traits he or she developed. Age and generational differences between the doctor and patient can also alter the relationship they develop. Moreover, the basic attitude of the doctor toward the patient may differ according to regional and historical differences in the professional education the doctor has received.

In sum, the individual DPR depends on multiple factors, including human individuality and chance. We do not know what our own mode is until we start forming a DPR. There is no one-size-fits-all DPR (Thomas, Best, and Mitchell 2020), as numerous and multidimensional variables are involved. It would be difficult to predict what DPR models will emerge in the future, as there are too many variables. The ideal DPR model may take on a cyclical pattern, and perhaps a paternalistic model will prevail in which AI decisions are broad-based (Asai et al. 2019).

## Complexity of the DPR

The DPR is neither pure nor simple; the components involved in the DPR are numerous and different in quality. In terms of how the DPR differs from ordinary personal relationships, the following are important examples. First, the DPR begins with a deliberate purpose, that is, the patient visits a healthcare organization in search of a solution to a problem. Second, to varying degrees, there is the issue of medical costs. Even with universal health insurance, patients pay 10 per cent to 30 per cent of the total medical cost, and medical expenses can influence the choice of treatment strategies. This is especially true for patients with limited financial resources. Third, friendship, emotional distance or closeness, and personal openness involved in the DPR can differ significantly. Fourth, the relationship is mainly limited to clinic hours within a single healthcare facility. Patient–doctor meetings outside of clinic hours away from the healthcare facility would be exceptional. Fifth, there is an imbalance of power due to differences in professional knowledge and experience, and physical and mental vulnerability. Sixth, the doctor needs to deal not only with the patient but also the patient’s family. The patient and family may have different intentions and wishes for medical care, making matters even more complex and unstable. Seventh, instead of a one-to-one relationship, the patient may be involved in a number of relationships with doctors treating the illness. Doctors are also engaged with multiple patients and have limited time to devote to a particular patient. Eighth, in most cases, neither party can choose the other at the time of the first visit or in an emergency. Finally, mutual trust is involved in the maintenance of a good relationship as well as good clinical outcomes. Thus, the DPR is highly individualized, can change over time, and is subject to social trends and chance, and hence, it is inherently complex.

## Confucian Doctrine of the Mean

This section discusses Confucius’ remarks on the Mean in *The Analects* and explains the Confucian Doctrine of the Mean. In the section that follows, we argue that, in the context of the diverse, ever-changing, and complex DPR, the attitudes and behaviours of doctors who have acquired the Mean would provide an appropriate platform for realizing the goals of healthcare. This mode of DPR is referred to as the doctor-seeking-the-Mean model.

In the *Kojien*, a leading Japanese dictionary, the Mean (*chūyō*) is explained as “to be unbiased and always unchanging,” “to be fair and impartial with no favoritism,” “not to be excessive or deficient,” “the middle way which is correct without bias,” “mediocrity,” and “to determine a middle ground between excess and deficiency with discretion (Aristotle’s concept of virtue)” (Niimura 2018, 1895). It is also used to mean “the middle way” or “a moderate and middle that does not run to extremes” (Kanaya 1993). It appears that the modern concept of the Mean intuitively emphasizes that it is just and correct to avoid extremes.

Confucius says in *The Analects,* “Supreme is the Mean as a virtue, but for a long time it has been rare among the people (Book 6-29)”; “To go beyond is no different from not arriving (Book 11-16)”; and “If one does not get hold of moderation (the Mean) to associate with, it is necessary to turn to either the impetuous or the cautious (Book 13-21)” (Confucius 1993). Confucius’ personality is also described by a disciple in *The Analects* as follows: “The master was genial and yet strict, imposing and yet not intimidating, courteous and yet at ease (Book 7-38)” and “The master cut out four things: he never took anything for granted, he never insisted on certainty, he was never inflexible, and never egotistical (Book 9–4)” (Confucius 1993). We believe that Confucius himself is the very embodiment of the Mean.

Confucius elaborates on the Mean in *the Doctrine of the Mean* (Confucius 2016). The Master said, “The superior man embodies the course of the Mean; the mean man acts contrary to the course of the Mean”; “Perfect is the virtue which is according to the Mean! Rare have they long been among the people, who could practice it!”; “The knowing goes beyond it (the path of the Mean) and the stupid do not come up to it … The men of talent and virtue go beyond it and the worthless do not come up to it.” Almost like *The Analects*, here too, it is stated that the superior person acts with the Mean, that the Mean is a perfect virtue, and that its practice is difficult (Confucius 2016). Then, Confucius refers to two characters, Shun and Hui, and describes their specific practices of the Mean:

There was Shun—Shun loved to question others, and to study their words, though they might be shallow. He concealed what was bad in them and displayed what was good. He took hold of their extremes, determined the Mean, and employed it in his government of the people … This was the manner of Hui—he made the choice of the Mean, and when he got hold of what was good, he clasped it firmly, as if wearing it on his breast and did not lose it. (Confucius 2016, 72)

Here it is shown that Shun did not discard extreme views but rather used the middle of the road, taking both ends of the spectrum into serious consideration. Referring to his disciple Hui, Confucius also states that a choice that has attained the Mean is a good one. According to Kanaya, a Japanese scholar of Chinese thought, the Mean is a virtue or attitude to life (Kanaya 1993). In Kanaya’s view, for Confucius, the Mean is a virtue or attitude that enables one to respond flexibly to changing circumstances and realize one’s goals without excess or deficiency; it is an attempt to fully understand all the diverse and conflicting views and positions, and then make the highest quality decision that best suits one’s need to achieve the goals at any given moment (Kanaya 1993). Kanaya also refers to Mencius’ statement that flexibility is also important to achieve the Mean, and that it is not good to be attached to one point, or to think in a fixed and immobile way. This is because things are constantly moving and are never stationary at both ends (Kanaya 1993). To achieve the Mean, it is important to be in the right place at the right time (Yahano 2016, 117).

Chinese researchers have argued that the Mean is the core concept of Confucianism and embodies a fundamental social principle as well as a cardinal human virtue (Chou 1979; Cheung et al. 2003), practically embracing the entire range of Confucian values and virtues. The Mean is often situation-specific, hence the emphasis is on the “timely mean” (Chou 1979; Cheung et al. 2003). The Mean also embraces the disposition to view oneself as embedded in a social system, and to see things holistically and in social terms, arguing that it is important to take another person’s goals into consideration as well as one’s own goals. The Mean seeks an appropriate point, i.e., the middle point, amidst a multitude of opposing forces so as to maintain social harmony in the interaction system. A major characteristic of the Mean is precisely that the empirical content of the Mean is not fixed and, hence, cannot be pre-defined (Cheung et al. 2003).

According to *The British Dictionary of Medical Ethics*, moderation (the Mean) means avoiding extremes and excess, reducing inflexibility, and acting reasonably. Its links with tradition may slow apparent progress but confer stability in an environment of change, pointing to different aspects of the Mean (Higgs 1997). The French philosopher Albert Camus discussed the value of the Mean in the section titled *the Mean and Extremism* in his 1951 book (Camus 1951). He thinks that there are limits to any thought, and that every thought or action beyond a certain point becomes self-denying. Camus also discusses that the Mean can resolve moral contradictions (antinomy), that achieving the Mean is a constant struggle, and that when virtue becomes too pure and detached from reality, it becomes evil. Moreover, he argues that, when discussing the value of the Mean, it is crucial to be realistic and avoid being overly idealistic. We believe that, for Camus, the Mean is an indispensable pragmatic principle and attitude to prevent the harmful effects of radical fundamentalism, absolutism, and idealism (Camus 1951). Taken together, we believe that the concept of the Mean includes the following seven elements: being goal-oriented, uniqueness, timely flexibility, comprehensiveness, humility, being realistic, and being difficult to achieve.

## On the Aims of the Confucian Doctrine of the Mean

As we shall see in the sections that follow, before arguing that the DPR compatible with the doctor-seeking-the-Mean model is the appropriate platform for realizing the goals of healthcare, we must be clear about the differences between these goals and the goal of the Confucian Mean. The Confucian Doctrine of the Mean assumes that Heaven and its normative function are real (Confucius 2016):

What Heaven has conferred is called the Nature; an accordance with this nature is called the Path of duty; the regulation of this path is called Instruction. (Confucius 2016, 71)

Sincerity is the way of Heaven. The attachment of sincerity is the way of men. He who possesses sincerity is he who, without an effort, hits what is right, and apprehends, without the exercise of thought—he is the sage who naturally and easily embodies the right way. He who attains to sincerity is he who chooses what is good, and firmly holds it fast. (Confucius 2016, 79)

Clearly, Confucius assumed that Heaven and its Way were real, and that the true nature bestowed upon human beings by the Way of Heaven was a good nature. The Way of Heaven is called “Sincerity.” Sincerity is not a human virtue but is considered the truth without falsehood and the original way of the providence and natural order of Heaven and Earth (Confucius 2016). The philosophy of the Mean is encompassed by the philosophy of Sincerity, and Sincerity is the foundation of the Mean (Kanaya 1993). The Mean is the virtue that man should acquire in order to realize his heaven-given good nature in life. In other words, the goal of the Mean is the embodiment of Sincerity, the Way of Heaven (i.e., objective goodness).

However, we are agnostic about the reality of normative heaven and the heavenly way and are also hesitant to say that human nature is good. We think that human beings have both good and evil parts within them, that there is in fact neither a perfect good individual nor perfect evil one, and that the theory of innate goodness of human beings and that of evil are extremes (Nozue 1998). We do not know the significance of human existence in the world or the purpose of human existence in the first place.

The aim of the Mean, as we describe it in this paper, differs from that of the Confucian Mean. Confucius taught the importance of practicing the Mean in order to realize Sincerity—the ultimate nature of Heaven. Meanwhile, we argue that a DPR compatible with the doctor-seeking-the-Mean model is an appropriate platform for realizing the purposes of medicine. The goals of healthcare that we aim to realize in the DPR are described in the next section.

## The Goal of the Doctor-Seeking-the-Mean Model

The purposes of medicine have been the subject of various and ongoing debates, differing in content and scope. Medicine is understood both as a science and an art and is a practice that contains numerous means in relation to the advancement of health. An exact definition of the concept of medicine is not forthcoming (Schramme 2017). But whatever the prevailing arguments about the purposes of medicine, people come to doctors for help, driven by the most urgent need for relief from physical and mental suffering and a return to health and normality (Williams 2007). In our view, life always has an aspect of suffering; it is full of the suffering of living, the suffering of aging, the suffering of illness, and despair for the extinction of which in the present life we are prescribing (Burtt 2000). Thus, suffering is a basic human experience. There are bodily, mental, social, and existential sufferings, and relieving them is considered a key goal for health professionals (Hofmann 2017).

The Hasting Centre set out in 1996 the goals of medicine as the prevention of disease and injury and the promotion and maintenance of health; the relief of pain and suffering caused by maladies; the care and cure of those with a malady and the care of those who cannot be cured; and the avoidance of premature death and the pursuit of a peaceful death (Hasting Center 1996). We agree that these are indeed the main goals of medicine. We believe that disease, injury, failure to maintain health of any kind, not being cared for, premature death, and failure of palliation at the end of life undoubtedly cause physical, psychological, and existential suffering in patients. Therefore, we consider the elimination or alleviation of patient suffering to be the most fundamental of the multiple goals of healthcare.

The DPR is a platform for the patient’s complaints of suffering and the doctor’s attempts to eliminate or alleviate it. Although it is unknown what the future holds, it is likely that the elimination and alleviation of patients’ health-related suffering remains the fundamental goal of medicine. Again, we do not know the significance or purpose of human existence, but we do realize that life is full of suffering and taking away human suffering is a goal of great value to us as human beings.

Understanding the extremes of a normative spectrum of judgements and issues, such as the spectrum of normative attitudes toward respecting patient self-determination and non-disclosure of serious illness diagnoses to patients, is important in pursuing the Mean in relation to these matters. However, as the breadth and content of this spectrum is subject to the constraints of the times and society, it should be recognized that this spectrum is of social construction. In other words, the spectrum used by the Mean is relative to the times and society. On the other hand, the absence of excess or deficiency that the Mean aims for is determined by the goal or destination. In this paper, we have set the destination as the realization of the goals of medicine, mainly the alleviation of patient suffering, which we believe is a universally important purpose that transcends differences in the times and society. Therefore, the doctor-seeking-the-Mean model that we propose can be said to be an activity that achieves this universal goal relative to the times and society.

## Usefulness of the Doctor-Seeking-the-Mean Model

Thus far, we have described the DPR as a complex relationship that is highly individualized, changeable over time, and is under the influence of social trends and chance. We have also elaborated on the Confucian Doctrine of the Mean, characterizing it by its goal-oriented nature, uniqueness, timely flexibility, comprehensiveness, humility, realistic nature, and difficult to achieve nature. The aim of the Mean, in our view, is the realization of the goals of medicine, which, in a nutshell, is the elimination or alleviation of patient suffering. In this section, we argue from various perspectives that the Confucian Doctrine of the Mean has utility in the DPR, which is highly individualized, changeable over time, diverse, complex, and susceptible to the social environment as well as chance.

First, it is almost impossible to unify the diverse modes of DPR—which is variable, highly individualized, complex, and susceptible to the environment of society, culture, and time, as well as chance—into a single, fixed, and specific mode. Trying to judge the appropriateness of individual DPRs based on a single ready-made model prevailing at the time also ignores reality. In particular, we believe that the idea of equating the DPR model with a decision-making model consisting of three main models ignores the complexity of the DPR, which must be considered based on a larger perspective than the decision-making model. The actual decision-making of a patient is strongly influenced not only by the power relations involved in decision-making but also by the patient’s psychology, finances, family, and chance.

The relationship with doctors that patients and their families seek to have may vary, with individual differences even within the same community or culture. The relationship between patients and their families is also individual-based. The patient-informed choice mode will not work in areas where patient autonomy is not a priority for both patients and their families. For patients who do not originally wish to participate in decision-making, the ideal SDM mode of relationship can be burdensome and even harmful. The idea that it is sufficient for doctors to automatically determine the DPR solely according to the patient’s wishes is problematic, as it is impossible to know whether or not the DPR model sought by the patient is in accordance with the medical aim of reducing the patient’s suffering without ascertaining the patient’s disease and psychological state. It must be taken into consideration where in the spectrum between the outcomes of the doctor’s respecting/ignoring patient desire the patient’s long-term interests lie.

For example, if a highly anxious patient asks the doctor for a family-like relationship, the relationship that is too emotionally close would lead the patient to have excessive expectations and make the patient overly dependent on the doctor, while also depriving the doctor of objectivity in clinical judgement (Ozeki-Hayashi and Wilkinson 2024). Conversely, a cold attitude from the doctor would lead to distrust and break the relationship. A doctor who has achieved the Mean would therefore express authentic but moderate friendliness. The degree of friendliness required here is not the middle or average of coldness and personal friendliness but is that which seems least harmful to the patient in the long term. However, a discussion of the relationship between the Mean and the emotional aspects of human beings is beyond the scope of this paper. When considering the emotions of doctors toward their patients in the DPR, it is necessary to consider the ways in which emotions are expressed, and the emotions themselves that arise spontaneously inside. In this paper, we are mainly concerned with the expression of emotions considered appropriate in the doctor-seeking-the-Mean model. Spontaneous emotions that arise in doctors toward their patients are not the subject of this discussion. In addition, while it is possible that a DPR that achieves the Mean would help gain the trust of patients more than a DPR that is extreme and unbalanced, addressing this point will require further exploration of the fundamental relationship between the concept of the Mean and human trust.

If a patient, who was doing well with the SDM model of DPR, becomes tired of fighting the disease or overwhelmed by the complexity of treatment, he or she may not need to be bound to continuing with patient-centred SDM. It would be acceptable to change the DPR and the decision-making mode flexibly according to the patient’s situation, sometimes with the family or doctor taking over the decision-making process. Also, for stubborn patients who are not willing to listen to the doctor’s opinion at all, the consumerist relationship may have to continue until the very last possible moment when it is not harmful to the patient. This is because the DPR itself will in many cases come to an end if the doctor is too eager to persuade such a patient. Nevertheless, the relationship must continue with patience and flexibility.

In cases of conflict between the patient and family regarding their wishes, it is practical to listen to both the patient and family and discuss their wishes; however, ultimately, the best policy for the patient should be sought, that is, to alleviate all kinds of suffering the patient is experiencing. The supremacy of the patient’s personal autonomy over the wishes of the family may not be desirable in view of future support that the patient may receive from the family members and their relationships. However, if the issue is the patient’s family’s request for non-disclosure of the diagnosis of a serious disease, the family should be persuaded to change their mind, unless there is evidence that the patient clearly wants non-disclosure. This is because, if the patient is unaware of a significant truth, it is often considered detrimental to the patient.

Confucius states, “To go beyond is no different from not arriving (Book 11-16),” which expresses the importance of goal setting and the problem of excess and deficiency (Confucius 1993). Although the basic goal of healthcare is to reduce patient suffering, a specific goal of patient care can be set differently depending on what the patient considers is the most significant suffering. For example, the goal of treatment for a patient who wants to continue treatment if there is room for improvement, no matter how painful, differs from that of a patient who does not want to undergo painful treatment, even if there is room for improvement; the point at which there is no excess or deficiency would naturally differ in the treatment of these patients. Suffering has a subjective element, and uniform goals cannot be set; therefore, it may be necessary to focus on the individuality of the patient’s goal in a timely and flexible manner.

In the event of an accidental encounter between a doctor and a patient with diametrically opposed medical beliefs, the harm caused to the patient and the doctor could be reduced if the doctor responds initially with an attitude of the Mean. Approaching the situation from the outset in a spirit of the Mean would, in our view, avoid the harm that fundamentalism, or extreme positions and attitudes, can cause. Patient distrust and discomfort in the event of a conflict can also be avoided. If one extreme position is criticized from the other, diametrically opposed position, a head-on collision may occur, resulting in rejection, confrontation, animosity, and conflict. As Camus pointed out, the Mean has the potential to resolve the moral dichotomy (Camus 1951).

In sum, to alleviate patient suffering, it is important for doctors to understand the particularity and individuality of the situation in question in the care of a particular patient in front of them, to maintain flexibility, to understand the extremes (spectrum) of the key issues of the DPR—a complex human relationship—to be familiar with their pros and cons, and to take realistic responses that integrate as many valuable perspectives as possible. We argue that the doctor-seeking-the-Mean model would enable this.

## Potential Ethical Issues and Difficulties That May Arise When the DPR of the Doctor-Seeking-the Mean Model is Introduced into Medical Practice

In this section, we predict issues that may arise when the Doctor-Seeking-the Mean model is introduced into medical practice in order to achieve reduced patient suffering as the ultimate goal of medicine. First, the goals of patient treatment and care will need to be determined more clearly than ever before. Whether or not an action or choice can achieve the Mean will be judged by whether or not it will achieve its goal. Therefore, the determination of treatment goals among those involved will become more important. However, strictly setting treatment goals for patients may lead to particularly difficult ethical conflicts. Thus, ethical discussions among those involved in the patient’s care will be required more than ever before.

Second, the Doctor-Seeking-the-Mean model does not align with pre-defined ethical guidelines or manuals. Simply following a set procedure will not be sufficient. When practicing medicine using our model, doctors must constantly check if their choices and actions are actually achieving the Mean, and this may increase the burden on doctors. As ethical education is expected to increase the virtue of doctors, the content of education must be improved to be more in-depth and fundamental, while ensuring that it resonates with the hearts of learners. Thus, it is necessary to educate learners to avoid misunderstanding the Mean simply as the product of compromise between the parties involved.

Third, the suffering of patients is diverse, and it has various aspects, such as mental, physical, existential, social, and economic aspects. In some cases, it is necessary to aim for the Mean in multiple dimensions simultaneously. It is undeniable that identifying the behaviour, attitude, or option that led to achieving the Mean may be difficult.

Finally, our approach based on the Doctor-Seeking-the Mean model will probably be criticized as compromising, opportunistic, or half-hearted by both fundamentalists or dogmatists—those who strongly believe in respecting patient autonomy and those who approve of paternalism on the part of doctors. In such cases, we will have to explain to them in detail that it is important to aim for the Mean in order to achieve the goals of medical care.

As Confucius says, the practice of the Mean is difficult to achieve. Likewise, there must be difficulties in implementing the Doctor-Seeking-the Mean model. However, we believe that compassionate doctors and medical institutions that put the interests of their patients first should spend time and energy on the practice and education of the Doctor-Seeking-the Mean model. More effort will be needed to achieve the best DPR that can alleviate the suffering of patients.

## Conclusion

We believe that the idea that the history of human society will improve linearly and that, eventually, a utopia will emerge for humans is overly optimistic. The same can be said of the healthcare system and the DPR. There are two sides to everything, i.e., advantages and disadvantages. All key concepts in medical ethics and bioethics also have two sides, and diverse DPR models are no exception (Mader 2004). Doctors’ assumption that all positions have a point of view makes it all the more important to have flexible and inclusive attitudes and behaviours in the DPR.

A deliberative model in which SDM takes place is not always the best for everyone. To establish a good DPR that contributes to the realization of the goals of medicine, instead of choosing one of the existing models, it is important to be flexible and adapt to the patient’s situation at the time. However, as Confucius states, the practice of the Mean may be difficult to achieve. Nevertheless, present-day doctors should approach the DPR with a routine attitude of “genial and yet strict, imposing and yet not intimidating, and courteous and yet at ease (Book 7-38).” One point to note is that the doctor-seeking-the-Mean model may coincide with existing models in some situations, and we hope to confirm that our model is not mutually exclusive with various other existing models. For example, for some patients, the doctor-seeking-the-Mean model may be a deliberative model.

Finally, like Confucius, Camus, and Aristotle, there are other contemporary scholars who refer to the practical importance of choices, attitudes, and lifestyles according to the Mean, being middle-of-the-road, balanced, and taking the line between conflicting extremes. Harari, a contemporary historian, argues that when secular people encounter ethical dilemmas, they do not ask, “What does God command?”; rather, they carefully weigh the feelings of all concerned parties, examine a wide range of observations and possibilities, and search for a middle path that will cause as little harm as possible (Harari 2018). Contemporary philosopher Weston also states, “To resolve practical moral questions and move ahead, we should try to balance and integrate various values at stake. When multiple values pull us to different directions on some practical questions, we work to find the choice that answers as many of the relevant values as fully as they can” (Weston 2021).

We conclude that the doctor-seeking-the-Mean model is important for the future of VUCA healthcare (Hamid 2019). Like Confucius in *The Analects*, when considering what a DPR achieving the Mean is like, doctors should never take anything for granted, never insist on certainty, never be inflexible, and never be egotistical (Book 9-4) (Confucius 1993).

## Data Availability

Not applicable because our study did not use any data or materials except published literature.

## References

[CR1] Ahuja, A. S. 2019. What is the best model of the physician–patient relationship? *Journal of Evaluation of Clinical Practice* 25 (6): 1111–1112. 10.1111/jep.13156 31037798

[CR2] Asai, A., T. Okita, A. Enzo, et al. 2019. Hope for the best and prepare for the worst: Ethical concerns related to the introduction of healthcare artificial intelligence. *Eubios Journal Asian and International Bioethics 2* (2): 64–70.

[CR3] Asai, A., T. Okita, and S. Bito. 2021. Considerations on points patients should pay attention to in the shared decision making process. *CBEL Report* 4 (1): 15–28.

[CR4] Asai, A., T. Okita, and S. Bito. 2022. Discussions on present Japanese psycho-cultural-social tendencies as obstacles toclinical shared decision-making in Japan. *Asian Bioethics Review* 14 (2): 133–150. 35069883 10.1007/s41649-021-00201-2 PMC8761963

[CR5] Burtt, E. A. 2000. *The teaching of the compassionate Buddha*. Penguin Publishing Group.

[CR6] Camus, A. 1951. *L’homme revolte*, 268–279. Trans: Sato Sand, and Shirai K. Shinchosha.

[CR7] Cartolovni, A., A. Malesevic, and L. Poslon. 2023. Critical analysis of the AI impact on the patient–physician relationship: A multi-stakeholder qualitative study. *Digital Health* 9:1–14. 10.1177/20552076231220833 PMC10734361 38130798

[CR8] Cheung, T. S., H. M. Chan, K. M. Chan, et al. 2003. On Zhongyong rationality: The Confucian doctrine of the mean as a missing link between instrumental rationality and communicative rationality. *Asian Journal of Social Science* 3 (1): 107–127.

[CR9] Childress, J. F., and M. D. Childress. 2020. What does the evolution from informed consent to shared decision making teach us about authority in healthcare? *AMA Journal of Ethics* 22 (5): E423–E429. 32449659 10.1001/amajethics.2020.423

[CR10] Chou, S. F. 1979. The doctrine of the mean and its impact on modern thought. *China Forum* 6:67–119.

[CR11] Confucius. 1993. *The Analects*. Trans: R. Dawson. Oxford: Oxford University Press.

[CR12] Confucius. 2016. *The complete Confucius*, 71–86. Trans: J. Legge. Melbourne: Golding Books.

[CR13] Emmanuel, E. J., and L. L. Emmanuel. 1992. Four models of the physician–patient relationship. *Journal of American Medical Association* 267 (16): 2221–2226. 1556799

[CR14] Hamid, D. S. 2019. The strategic position of human resource management for creating sustainable competitive advantage in the VUCA World. *Journal of Human Resources Management and Labor Study* 7 (2): 1–4.

[CR15] Harari, Y. N. 2018. *21 Lessons for the 21st century*. Vintage.

[CR16] Hasting Center. 1996. Special supplement: The goals of medicine: setting new priorities. *Hasting Center Report* 26 (6): S1–S27. 8970793

[CR17] Hayashi, R. O., D. and J. C. Wilkinson. 2024. Shinmi: A distinctive Japanese medical virtue? *Asian Bioethics Review* 16:563–573. 39398457 10.1007/s41649-023-00261-6 PMC11465113

[CR18] Higgs, R. 1997. Moderation. In *The New Dictionary of Medical Ethics*, eds. K. M. Boyd, R. Higgs, and A. Pinching. London: BMJ Publishing Group.

[CR19] Higuchi, N. 1998. The right of patients to self-determination. In *Contemporary Law 14*, ed. I. Shoten, 75. Tokyo: Iwanami Shoten.

[CR20] Hofmann, B. 2017. Suffering: Harm to bodies, minds, and persons. In *Handbook of the philosophy of Medicine*, eds. T. Schramme and T. Edwards, 129–145. Dordrecht: Springer.

[CR21] Japan Medical Association Research Institute for Policy and Practice. 2017. The 6th survey on attitudes towards medical care in Japan. In *Nihon Iyaku Soken Working Paper*. No. 384: 76.

[CR22] Kanaya, O. 1993. *Thinking about Chinese thought: Tradition that opens the future*. Tokyo: Chuokoron-shinsho.

[CR23] Kanaya, O. 1998. Commentary. In *The Great Learning and the Doctrine of the Mean, Confucius*. Trans. O. Kanaya, 125–138. Tokyo: Iwanami Shoten.

[CR24] Kellerman, P. S. 2018. Old friends: Maintaining the physician–patient connection in the 21st century. *American Journal of Kinney Disease* 71 (3): 12–13. 10.1053/j.ajkd.2017.09.028 29290479

[CR25] Levinson, W., A. Kao, A. Kuby, et al. 2005. Not all patients want to participate in decision making. A National study of public preferences. *Journal of General Internal Medicine* 20:531–535. 15987329 10.1111/j.1525-1497.2005.04101.x PMC1490136

[CR26] Lo, B. 2020. *Resolving ethical dilemmas: A guide for clinicians*. 6th edition. Philadelphia: Wolters Kluwer.

[CR27] Loxterkamp, D. 2023. What humans need. *Annals of Family Medicine* 21 (5): 465–467. 37748911 10.1370/afm.3015 PMC10519765

[CR28] Mader, S. S. 2004. *Human Biology*. 8th edition. Boston: McGraw Hill.

[CR29] Murray, E., L. Pollack, and M. White, et al. 2007. Clinical decision making: Patients’ preferences and experiences. *Patient Education and Counseling* 65 (2): 189–196. 16956742 10.1016/j.pec.2006.07.007

[CR30] Niimura, I. 2018. *Kojien [Japanese dictionary]* Vol. 2, 7th edition. Iwanami Shoten.

[CR31] Nozue, C. 1998. Foreword by the Supervisor. In *Manga: The thought of Mencius, the Great Learning and the Doctrine of the Mean* (illustrations and words by T. C. Chung, edited by C. Nozue, translated by T. Wada). Tokyo: Kodansha.

[CR32] Schramme, T. 2017. Goals of Medicine. In *Handbook of the philosophy of Medicine*, eds. T. Schramme and T. Edwards, 121–128. Springer.

[CR33] Suh, J. 2020. The Confucian doctrine of the Mean, the optimality principle, and social harmony. *Society and Economy* 42 (1): 59–73.

[CR34] Szasz, T., and M. Hollender. 1956. A contribution to the philosophy of medicine: The basic model of the doctor–patient relationship. *Archives of Internal Medicine* 97 (5): 585–592. 13312700 10.1001/archinte.1956.00250230079008

[CR35] Tamblyn, N. 2016. Introduction. In *The complete Confucius, Confucius*. Trans: J. Legge, i–iii. With an introduction by Nicholas Tamblyn. Melbourne: Golding Books.

[CR36] Thomas, H., M. Best, and G. Mitchell. 2020. Whole-person care in general practice: The doctor–patient relationship. *Australian Journal of General Practitioners* 49 (3): 139–144. 10.31128/AJGP-05-19-49502 32113208

[CR37] Veatch, R. M. 1972. Models for ethical medicine in a revolutionary Age. *Hasting Center Report* 2 (3): 5–7. 4679693

[CR38] Weston, A. 2021. *A practical companion to ethics* 5th edition. Oxford University Press.

[CR39] Williams, J. R. 2007. *World Medical Association Medical Ethics Manual*. World Medical Association, Cedex, France, 2005. Japanese translation published by the Japan Medical Association, Norio Higuchi edition, Tokyo.

[CR40] Xu, H., R. Yining, T. Okita, et al. 2024. Reflections from Chinese and Japanese physicians on medical disputes. *Asian Bioethics Review* 16:683–709. 39398454 10.1007/s41649-024-00294-5 PMC11464652

[CR41] Yahano, T. 2016. *The great learning and doctrine of the mean*. Tokyo: Kadokawa Sophia Bunko.

[CR42] Yoneura, Y. 2023. *Medical Law Lecture*. 2nd edition. Nippon Hyoronsha.

[CR43] Yoshida, S. 2020. Decision-making support for patients. *Japan Journal of Behavioral Medicine* 25 (6): 161–163.

